# Intestinal Microbiota as an Alternative Therapeutic Target for Epilepsy

**DOI:** 10.1155/2016/9032809

**Published:** 2016-11-01

**Authors:** Jiaying Wu, Yuyu Zhang, Hongyu Yang, Yuefeng Rao, Jing Miao, Xiaoyang Lu

**Affiliations:** ^1^The First Affiliated Hospital, College of Medicine, Zhejiang University, Hangzhou, China; ^2^Ningbo Medical Treatment Center Lihuili Hospital, Ningbo, China

## Abstract

Epilepsy is one of the most widespread serious neurological disorders, and an aetiological explanation has not been fully identified. In recent decades, a growing body of evidence has highlighted the influential role of autoimmune mechanisms in the progression of epilepsy. The hygiene hypothesis draws people's attention to the association between gut microbes and the onset of multiple immune disorders. It is also believed that, in addition to influencing digestive system function, symbiotic microbiota can bidirectionally and reversibly impact the programming of extraintestinal pathogenic immune responses during autoimmunity. Herein, we investigate the concept that the diversity of parasitifer sensitivity to commensal microbes and the specific constitution of the intestinal microbiota might impact host susceptibility to epilepsy through promotion of Th17 cell populations in the central nervous system (CNS).

## 1. Introduction

Epilepsy is one of the most common severe neurological diseases and a nonnegligible cause of handicap and mortality. Based on epidemiological data, there are currently more than 70 million epileptic patients in the world, and the number is expected to increase gradually in the future [1, 2]. Even though there has been decades of extensive investment in clinical and scientific research, the etiology of epilepsy is still unclear [3, 4]. Dozens of studies have confirmed the genetic basis for diverse epileptic seizures [5–7]. However, the data from a multicenter twin collaboration do not reveal any similarity between twin pairs with classic benign rolandic epilepsy [8], indicating that noninherited environmental factors might also play a crucial role in the progression of epilepsy.

Antiepileptic drugs (AEDs) are the main feasible therapeutic methods used to treat epilepsy. At present, there are only 20 AEDs licensed globally, and there are many disadvantages associated with their use. Apart from the inevitable adverse effects, AEDs only restrain the symptoms of seizure rather than modify the development of epilepsy. Furthermore, AEDs are only regarded as effective in 60–70% of epileptic individuals [2, 4]. In consideration of the limited numbers of therapeutic treatments and the poor reactivity to available AEDs, it is urgent to deeply clarify the underlying etiologies of epilepsy for developing new therapeutic strategies.

Autoimmune processes and associated pathogenic autoantibodies have recently received increasing attention for their role in idiopathic seizure disorders [9–12]. A population-level epidemiological study documented that epilepsy and some autoimmune diseases frequently co-occur, and the potential role of autoimmunity in epilepsy must be given due consideration [13]. Of great interest are the many species of intestinal microbes that reside in great numbers in the digestive tract. These gut microbiota may play a vital role not only in the maintenance of microbiota homeostasis and food digestion, but also in the progression of autoimmune diseases through modulation of immune responses. Paradoxically, some species of intestinal microbes seem to potentiate diseases, whereas others may prevent diseases [14–16]. For example, replenishment of probiotics such as the phyla Firmicutes and Bacteroidetes may be useful in preventing type 1 diabetes (T1D) [16, 17]. On the other side, intestinal colonization with* segmented filamentous bacteria* (SFB) tends to increase the risk of experimental autoimmune encephalomyelitis (EAE), an animal model for multiple sclerosis (MS), and autoimmune arthritis [18, 19]. Therefore, in view of the robust association between epilepsy and autoimmunity, it is quite possible that the community composition of the intestinal microbiota could affect host susceptibility to epilepsy and further modulate disease progression.

## 2. Incidence of Epilepsy

Epilepsy is a common disorder that probably has a complicated correlation with environmental factors. Across the globe, more than 85% of epileptic patients live in poverty-stricken areas that have 49% of the world's population [2], so it is possible that poor hygiene is a nonnegligible cause of epileptic attacks. Furthermore, the majority of epileptic sufferers in developing territories have little chance to receive standard and long-term antiepileptic therapy [20]. Therefore, exploring new etiological strategies to modify epilepsy predisposition and decrease the risk of epilepsy may have more practical importance than relying on traditional medication to control symptoms.

## 3. Autoimmunity and Epileptogenesis

The association of hygiene with epilepsy led to the assumption that epilepsy could be an immune-related disorder. In spite of the facts that many immune disorders (e.g., T1D, inflammatory bowel disease, lupus, and asthma) are closely linked with greater hygiene, it cannot be excluded that many factors other than hygiene are also related with these immune disorders. The immune system is indispensable for self-protection and recovery from multiple infectious or noninfectious inflammations and insults. The central nervous system (CNS) is regarded as an immunologically specialized site, which evolved into two independent but related parts: the innate immune system, which is responsible for the immediate response against external stimulation, and the adaptive immune system, in which antigens are recognized by specific antigen-presenting cells and antigen receptors [21]. The innate immune system is composed of mast cells and phagocytic cells comprising monocytes/macrophages and microglia with a highly conservative class of pattern-recognition receptors, for instance, toll-like receptors (TLRs), whereas the adaptive immune system mainly consists of T and B lymphocytes that have diverse receptor systems [22, 23].

Autoimmune diseases result from an aberrant immune response of the body against its own substances and tissues. The pathogenesis of autoimmune diseases has still not been determined, although factors such as the environment, medicine, diet, genetics, and immune system are all deemed important incentives. Generally speaking, autoimmunity usually occurs without a clinical symptom only if the extra forces such as surrounding changes cause the overt expression [23, 24]. For a long time recognition of self-antigens by the adaptive immune system has been regarded to be the core mechanism of autoimmune disorder pathogenicity, but recently attention has focused on the role of innate immune system [23].

It has been verified that a substantial number of epilepsy cases have an autoimmune-related basis, and for these cases adjunctive immunotherapy was effective in slowing, halting, or even reversing the development of epilepsy [10]. Moreover, a few observational studies have demonstrated that epileptic patients might benefit from specific immunotherapy to control seizures [25–28]. A number of studies have reported that several serum autoantibodies are epileptogenic, and immunomodulation might effectively alleviate the development of some epilepsy syndromes [27–29]. Autoantibody specificities recognized in the setting of epilepsy include, for example, voltage-gated potassium channel complex, N-methyl-D-aspartate receptor [30], glutamic acid decarboxylase 65 [27], and *γ*-aminobutyric acid B [11, 31]. Recently, a retrospective population-based epidemiological study confirmed an association between the prevalence of epilepsy and the autoimmune process [13]. The research focused on 12 major autoimmune diseases, and all of these autoimmune diseases were found to be correlated with a high predisposition for epilepsy. Among these diseases, antiphospholipid syndrome, systemic lupus erythematosus, and T1D had the strongest links [13]. The prevalence of epilepsy is 0.4% in a normal population but increases to 17.5% among autoimmune disease patients [11]. Moreover, observational studies have demonstrated that epileptic patients might also benefit from specific immunotherapy to control seizures [25–28], which further highlights the potential role of autoimmunity in epilepsy.

## 4. Expanding the Role of Intestinal Microbiota in Autoimmune Epilepsy

### 4.1. Interactions between Intestinal Microbiota and Host Immunity

The intestinal microbiota is a complex community of trillions of microorganisms inhabiting the digestive tracts of animals with various dynamic interactions with the host immune system. Gut microbes benefit the host in many ways, such as improving digestion, nourishing the intestinal epithelia, and suppressing pathogenic microbial growth [32]. The relationship between the gut microbiota and the host organism is not just simply commensal, but rather a mutualistic symbiosis [33]. It has been shown that changing the communities of gut microbes in newborns by delivering beneficial microbes can influence infant growth and future health [34].

### 4.2. Intervention by Exposure to Intestinal Microbes during the Course of Autoimmune Diseases

The gut microbiota and the immune system are closely connected and simultaneously influence each other [35]. Immune modulation by the microbiota is not only restricted to the intestinal environment, but also influences other peripheral organs [32, 36]. Given this tight linkage, it is not surprising that gut-residing bacteria are involved in the pathogenicity of autoimmune disorders. An interesting study indicated that the patients with Crohn's disease or ulcerative colitis presented abnormal intestinal microbiota, characterized by depletion of two bacterial genera—the phyla Firmicutes and the Bacteroidetes [14]. This dysbiosis is significantly associated with the disease phenotype and may be used as a biomarker for the diagnosis of Crohn's disease [15]. Intriguingly, these two bacterial taxa can also ameliorate the progression of nongut autoimmune diseases such as T1D [16]. Germ-free nonobese diabetic mice with a knockout mutation in an adaptor protein involved in the TLR and IL-1 receptor innate immune response signaling pathway (MyD88^KO^) suffered robust T1D, whereas inoculation of these mice with an established microbial community (representing natural bacterial phyla present in the human digestive tract) decreased the morbidity of T1D [16]. High-throughput, culture-independent approaches also identified species of microbes that are involved in the development of T1D-related autoimmune responses in young children and therefore could be used as microbial markers for early diagnosis of T1D [17]. The lack of four different bacterial taxa,* Lachnospira*,* Veillonella*,* Faecalibacterium*, and* Rothia*, in newborn infants was reported to be a cause of high susceptibility to childhood asthma [37]. Together, these examples demonstrate the promise of microecosystem-based diagnostics and therapies in preventing the development of immune-related diseases.

However, the gut microbiota is not always a friend of humans in the control of autoimmune disorders. Microbes are a double-edged sword to the host and have been implicated in the regulation of both pro- and anti-inflammatory immune responses [38–40]. In contrast to T1D, where germ-free conditions exacerbate disease symptoms, the morbidity of autoimmune arthritis is much lower in K/BxN mice reared in germ-free conditions. A germ-free feeding environment also leads to a reduction in serum autoantibody titers and the splenic T helper 17 (Th17) cell population. Th17 cells are a subset of proinflammatory T helper cells and contribute to pathogen clearance at mucosal surfaces. The loss of Th17 cell populations has been linked to chronic inflammation and microbial translocation [41]. SFB, a gut-residing species, was capable of driving arthritis development via promoting an increase in Th17 cell populations [19]. SFB is also a critical factor in the onset of MS, which is a serious autoimmune disease characterized by progressive deterioration of neurological function [18]. Mice raised in a sterile environment developed strikingly decreased EAE, an animal model for MS, compared with controls. Germ-free rodents harboring SFBs alone effectively stimulated proinflammatory T-cell responses and developed EAE, suggesting that certain intestinal bacteria could drive neurologic inflammation [18]. In a randomized controlled trial, hypokinesia in Parkinson's disease patients was alleviated after the eradication of* Helicobacter pylori* [42]. It is quite interesting that different species of the gastrointestinal microbiota have diverse effects on the development of different autoimmune diseases. Based on the data above, it seems that the members of the phyla Firmicutes (e.g., Lactobacillaceae) and Bacteroidetes (e.g., Rikenellaceae and Porphyromonadaceae) may be therapeutical against certain autoimmune disorders via interaction with the innate immune system. In contrast, SFB plays a negative role through its impact on the adaptive immune system. Therefore, it is highly important to clarify the role of intestinal microbiota in directing host immune responses in the design of logical therapies for diverse autoimmune disorders.

### 4.3. Correlation between Intestinal Microbiota and Epilepsy

Although the effects of gut bacteria on autoimmunity may be different in distinct pathological processes and the mechanisms of induction of local or systemic immunoreaction by microbiota remain to be illuminated, recent studies have highlighted a critical role of intestinal microbiota in the development of autoimmune diseases. Taking into account the close relationship between epileptogenesis and autoimmune diseases, it is quite possible that intestinal microbiota are able to alter the progression of epilepsy. Epidemiological statistics indicate that the incidence of epilepsy is 45.0/100,000/year (interquartile range (IQR) 30.3–66.7) for developed countries and 81.7/100,000/year (IQR: 28.0–239.5) for developing countries [2]. For unclear reasons, even in developed countries, there is always a higher incidence of epilepsy among people living in low- and middle-income areas [43]. Considering the lack of sanitation in low-income areas, we strongly suspect that the exposure to certain gut bacteria such as SFBs could be responsible for epilepsy prevalence in resource-poor countries.

## 5. Intestinal Microbiota Regulates Proinflammatory T-Cell Responses in Epileptogenesis

Intracerebral inflammatory reactions may contribute to the pathophysiological lesions observed in epileptic and seizure disorders [44]. The levels of proinflammatory cytokines such as interleukin 6 (IL-6) and interferon *γ* in the peripheral blood of the epileptic patients are usually higher than those of control patients, implicating the role of immune factors in the disease process of epilepsy [45]. Recently, it was reported that the level of IL-17A, either in the cerebrospinal fluid (CSF) or in the peripheral blood of patients with epilepsy, was significantly elevated and the level of IL-17A was highly correlated with seizure frequency and severity [46]. IL-17A is the canonical cytokine produced by the helper T (Th) cell 17 subset of lymphocytes. Th17 cells have been demonstrated to be involved in inflammatory responses in many autoimmune diseases that impact the nervous system [47], highlighting the key role for Th17/IL-17A signaling in the pathogenesis of seizure disorders.

The commensal bacteria in our gastrointestinal tract possess the ability to activate the proinflammatory Th cells in a controllable way without inducing extensive pathological inflammation, but sometimes they may also drive our self-immune system to autoimmunity [18, 48]. Proinflammatory CD4^+^ T cells, which are pivotal components of the adaptive immune system, generally consist of three subtypes, including Th1, Th2, and Th17 cells. Of these, Th17 cells have recently been shown to be modulated by the symbiotic gut bacteria [49]. Th17 cell differentiation was directed by specific subsets of bacteria such as Bacteroidetes in a dynamic and reversible manner [50]. Furthermore, germ-free mice have a deficiency of splenic IL-17-producing T cells [19], and the intestinal dendritic cells of germ-free rodents also had a decreased ability to trigger proinflammatory T-cell responses [18]. Gut colonization with a single type of microbiota, SFB, was specifically capable of inducing Th17 cell differentiation and promoting IL-17 production either in the intestines or in other organs [48, 51]. It was reported that SFB triggered autoimmune arthritis development in K/BxN mice via promotion of lamina propria Th17 cell compartment, leading to high production of arthritis-associated autoantibodies [19]. During the induction of EAE, SFB exposure induced Th17 cell in the CNS, impacted the balance between pro- and anti-inflammatory immune responses, and then promoted the development of EAE [18]. Moreover, Th17 cells also provided special route of gut-lung interaction in allergic airway disease. Increased IL-17A levels in the bronchoalveolar lavage fluid and enhanced Th17 cell numbers in the spleen were detected in old allergic mice whose microbial community structure was quite different from that of young and healthy ones, indicating a close association between the emergence of Th17 cells and the development of allergic asthma [36]. Thus, it is reasonable to hypothesize that IL-17A-producing CD4^+^ Th17 cells might play a critical role in epileptogenesis after exposure to certain commensal bacteria.

Although there is a close connection between SFB and Th17 cells, it should be noticed that SFB are indigenous gut microbiota of rodents, fish, and chickens. In human, SFB colonization occurs within the first 2 years of life and declines in an age-dependent manner [52]. Are there any other human commensal microorganisms capable of exerting immunological effects equivalent to those of SFB? In a recent study, inoculation of human fecal samples from patients with ulcerative colitis to germ-free mice did exhibit a significant increase in Th17 subset in colon [53]. After analysis of the Th17-inducing bacterial species, 20 strains from diverse species were isolated, including* Clostridium*,* Bifidobacterium*,* Ruminococcus*, and* Bacteroides*. Furthermore, the adhesion of microbes to intestinal epithelial cells may be a critical cue for Th17 cell induction [53].

How can commensal microbes influence the production of IL-17 by Th17 cells? Experimental studies have shown that cytokine production is significantly associated with epileptogenesis. Levels of IL-6 mRNA were increased within the hippocampi of rats with epilepsy, and IL-6 was also involved during the process of kindling [54]. Higher CSF/serum IL-1*β* ratios were correlated with an increasing risk for diverse types of epilepsy [55]. A meta-analysis further showed that two alleles of proinflammatory cytokines (IL-1*α*-889 and IL-1*β*-511) and the serum concentration of IL-6 were significantly associated with epilepsy [56, 57]. Moreover, it is well established that proinflammatory cytokines, in addition to their canonical involvement in the mechanisms of seizure generation, have a crucial role in driving and modulating human Th17 responses. The monocytes from T1D subjects spontaneously secreted more IL-6 and IL-1*β*, which are known to induce and expand Th17 cells compared with those from healthy control subjects [58]. Considering the high cooccurrence of epilepsy and T1D [12, 59], it is possible that the commensal microbiota drive autoimmune epileptogenesis through expansion of Th17 cells mediated by spontaneous secretion of proinflammatory cytokines, for instance, IL-6 and IL-1*β*.

## 6. Conclusion

Various studies have demonstrated that intestinal microbiota influence gut-brain immune responses during the progression of diverse autoimmune diseases. There is increasing evidence that the pathological mechanisms of autoimmunity might also play an important role in epilepsy. In this paper, we have summarized the current relevant literature and have tried to bridge the gap between experimental and clinical evidence to discuss the possible cause-and-effect relationship between intestinal microbiota and epilepsy. We speculate that dysbacteriosis of the gastrointestinal microorganisms may be an important factor in the development and/or severity of epilepsy and that immune-stimulation by the microbiota could provide an alternative strategy for treatment of inflammation-related diseases such as epilepsy.
